# Plasma hPG_80_ (Circulating Progastrin) as a Novel Prognostic Biomarker for Hepatocellular Carcinoma

**DOI:** 10.3390/cancers14020402

**Published:** 2022-01-13

**Authors:** Marie Dupuy, Sarah Iltache, Benjamin Rivière, Alexandre Prieur, George Philippe Pageaux, José Ursic Bedoya, Stéphanie Faure, Heloïse Guillaumée, Eric Assenat

**Affiliations:** 1 Department of Medical Oncology, CNRS UMR 5535 St-Eloi University Hospital Montpellier, School of Medicine, 34295 Montpellier, France; m-dupuy@chu-montpellier.fr (M.D.); sarah.iltache@gmail.com (S.I.); b-riviere@chu-montpellier.fr (B.R.); gp-pageaux@chu-montpellier.fr (G.P.P.); j-ursicbedoya@chu-montpellier.fr (J.U.B.); s-faure@chu-montpellier.fr (S.F.); h-guillaumee@chu-montpellier.fr (H.G.); 2 ECS-Progastrin, 1004 Lausanne, Switzerland; a.prieur@ecs-progastrin.com

**Keywords:** hepatocellular carcinoma (HCC), hPG_80_, circulating progastrin, blood-based biomarker, prognostic value

## Abstract

**Simple Summary:**

Liver cancer is the sixth most common cancer world-wide and hepatocellular carcinoma (HCC), the most common form of primary liver cancer, accounts for 90% of the cases. The diagnosis of HCC is usually based on non-invasive criteria using detection of a liver nodule in abdominal ultrasonography or high serum alpha-fetoprotein (AFP) levels. However, as it is only elevated in 60% of patients with HCC, AFP has limited accuracy, especially in early stages, as both a diagnostic and prognostic test. We investigated hPG80 (circulating progastrin), which is associated with liver cancer biology, and found that hPG80 levels is both an independent prognostic marker in HCC and used in combination with AFP, it improves the stratification of the patients in good and poor prognosis, especially for those patients at early-stage. This will help stratify HCC patients more accurately in the future and improve the management of these patients.

**Abstract:**

Alpha-fetoprotein (AFP) is the most widely used biomarker for hepatocellular carcinoma (HCC) prognosis. However, AFP is not useful in establishing a prognosis for patients with a tumor in the early stages. hPG_80_ (circulating progastrin) is a tumor promoting peptide present in the blood of patients with various cancers, including HCC. In this study, we evaluated the prognostic value of plasma hPG_80_ in patients with HCC, alone or in combination with AFP. A total of 168 HCC patients were tested prospectively for hPG_80_ and analyzed retrospectively. The prognostic impact of hPG_80_ and AFP levels on patient survival was assessed using Kaplan-Meier curves and log-rank tests. hPG_80_ was detected in 84% of HCC patients. There was no correlation between hPG_80_ and AFP levels in the training and validation cohorts. Both cohorts showed higher sensitivity of hPG_80_ compared to AFP, especially at early stages. Patients with high hPG_80_ (hPG_80_+) levels (optimal cutoff value 4.5 pM) had significantly lower median overall survival (OS) compared to patients with low hPG_80_ (hPG_80_−) levels (12.4 months versus not reached respectively, *p* < 0.0001). Further stratification by combining hPG_80_ and AFP levels (cutoff 100 ng/mL) improved prognosis in particular for those patients with low AFP level (hPG_80_−/AFP+ and hPG_80_−/AFP−, 13.4 months versus not reached respectively, *p* < 0.0001 and hPG_80_+/AFP+ and hPG_80_+/AFP−, 5.7 versus 26 months respectively, *p* < 0.0001). This was corroborated when analyses were performed using the BCLC staging especially at early stages. Our findings show that hPG_80_ could serve as a new prognostic biomarker in HCC. Used in combination with AFP, it improves the stratification of the patients in good and poor prognosis, especially for those patients with negative AFP and early-stage HCC.

## 1. Introduction

Liver cancer is the sixth most common cancer world-wide, with 841,080 new liver cancer cases in 2018, and the fourth leading cause of cancer-related death globally [1]. It is estimated that, by 2025, over a million individuals will be affected by liver cancer annually [2]. With a five-year survival of 18%, liver cancer is the second most lethal tumor after pancreatic cancer [3]. Hepatocellular carcinoma (HCC), the most common form of primary liver cancer, accounts for 90% of the cases. Typically, HCC develops on a background of advanced chronic liver disease, with HBV infection, HCV infection, alcohol abuse and nonalcoholic fatty liver disease being the major etiologies [4].

The management of HCC has substantially improved over the past decade. The treatment is assigned according to tumor stages and the expected benefits of major interventions, following the Barcelona Clinic Liver Cancer (BCLC) staging system [5]. In principle, patients with early stage HCC tumors are the preferred candidates for resection, transplantation, and local ablation, whereas patients at intermediate stages are first candidates for transarterial chemoembolization (TACE) and those with advanced disease will first receive systemic therapies. The median survival times for early, intermediate, and advanced HCC are 5 years, 2.5 years and 10 months, respectively [6]. There is a real need to have a good tool to diagnose the patient as early as possible.

The diagnosis of HCC is usually based on non-invasive criteria using detection of a liver nodule in abdominal ultrasonography or high serum alpha-fetoprotein (AFP) levels [7]. Although a non-invasive diagnosis, i.e., based on imaging and AFP, is allowed when a typical image finding occurs during cirrhosis, surveillance and a biopsy confirmation is often needed for HCC diagnosis. However, as it is only elevated in 60% of patients with HCC (even less at early stages), the most widely used biomarker, AFP, has limited accuracy, especially in early stages, as both a diagnostic and prognostic test [8].

We have reported previously of hPG_80_ (human circulating progastrin) being elevated in the plasma of patients with various types of cancers [9]. In physiology, progastrin is the precursor of gastrin synthetized by antrum G cells and processed into gastrin [10]. Progastrin does not accumulate in G cells, by contrast to G34-Gly and gastrin [11]. G34-Gly will generate gastrin upon full maturation. As a consequence, progastrin is barely detectable in the blood of healthy subjects, even though few of them have been shown to be positive as observed the first time by Siddheshwar et al. [12]. However, in line with the demonstration of the expression of the *GAST* gene, encoding progastrin, in colorectal tumors as well as other tumor types, high levels of hPG_80_ (named as such when progastrin is released from tumor cells and detected in the blood) were reported in the blood of cancer patients [9,12,13,14]. Moreover, in addition to the fact that *GAST* is a direct target of the ß-catenin/Tcf4 pathway, activated in many cancers, including HCC [15], a large body of literature supports the functional role of hPG_80_ in tumorigenesis [14,16,17,18,19,20]. As a consequence, hPG_80_ is an interesting indicator of tumor behavior/activity and clinical outcomes.

In the present study, we determined the prognostic performances of hPG_80_ in clinically diagnosed HCC patients, alone or in combination with AFP, and examined whether hPG_80_ might improve the stratification of patients to predict overall survival (OS), taking into account the BCLC staging or not.

## 2. Materials and Methods

### 2.1. HCC Patients

A total of 168 HCC patients managed with local or systemic treatments, including molecular targeted agents (“Liverpool” biobank) were tested prospectively for hPG_80_ and analyzed retrospectively (Table 1). All patients provided written consent for research at the time of their blood collection, in line with international regulations and the ICH GCP (International Conference on Harmonization- Good Clinical Practice) guidelines (N° 2019_IRB-MTP_01-11). All patients received clinical diagnostics, and 94 patients received histological diagnostics (biopsy or surgical specimen), with material usable for immunohistochemical analyses.

For the training cohort, we used the first 84 patients enrolled in this cohort between July 2015 and December 2017. For the validation cohort, we used the last 84 patients enrolled in this cohort between February 2018 and October 2019.

For the prognosis value analysis, we pooled all the 168 HCC patients from the training and validation cohorts.

### 2.2. hPG_80_ Level Measurements in the Blood Samples

The ELISA DxPG_80_.lab kit (ECS-Progastrin, Lausanne, Switzerland) was used to measure hPG_80_ levels in all plasma EDTA samples according to the manufacturer’s instruction. The analytical performances of the kit are described in Cappellini et al. [21]. Briefly, the limit of detection (LoD) is at a hPG_80_ concentration of 1 pM and the limit of quantitation (LoQ) is at a hPG_80_ concentration of 3.3 pM. The inter- and intra-assay coefficients of variation (CV%) is below 10%. No cross-reactivity was detected with gastrin-17, Gastrin-Gly or CTFP (C-Terminus Flanking Peptide). No cross-reactivity was detected with other blood biomarkers such as CA125, CEA or PSA. No interference was detected with chemicals such as SN-38, 5-FU or triglycerides, cholesterol or hemoglobin.

### 2.3. AFP Level Measurements in the Blood Samples

The blood-based biomarker AFP (alpha-fetoprotein) concentrations were centrally measured using Cobas E411 (Roche Diagnostic France, Meylan, France) with Elecsys AFP (Roche Diagnostic France, Meylan, France).

### 2.4. Immunohistochemical Analyses

HCC specimens collected from patients who had undergone liver biopsy were formalin-fixed and paraffin-embedded (FFPE). In each HCC specimen, the histological subtypes [22] and grades were determined according to the WHO classification by an expert liver pathologist (B.R.).

The following primary antibodies were used: anti-EpCAM (clone Ber-EP4, Dako, Agilent technology France, Les Ulis, France), anti-CK19 (clone RCK108, Dako, Agilent technology France, Les Ulis, France), anti-glutamine synthetase (GS, clone 6/GS, BD Biosciences, Le Pont de Claix, France) and anti-p53 (clone DO-7, Roche/Ventana). Immunohistochemical assays were performed on the Ventana Benchmark Ultra automated staining instrument (Ventana Medical Systems, Illkirch, France). For each antibody, staining was analysed using the following scores: surface (0: 0–5%, 1: 5–25%, 2: 25–50% and 3: >50%) and intensity (0: no expression, 1: weak expression and 2: high expression). Stainings were considered positive if the percentage of tumor cells was >5%.

### 2.5. Statistical Analysis

An optimal cutoff value of hPG_80_ was defined using the function of “surv_cutpoint” in R Package “survminer”, calculating the minimal *p*-value based on the log-rank method. R software 3.6.1 (The R Foundation for Statistical Computing) was used. The overall survival (OS) was defined as the elapsed time from blood collection to date of death or end of the study. The survival curves were constructed using the Kaplan-Meier method and compared performing a log-rank test on HCC patients. Clinicopathological variables between patients with low and high hPG_80_ or AFP levels were compared using the Fisher’s exact test. Correlations between hPG_80_ and AFP, MELD, creatinine levels and age were evaluated using the Spearman correlation coefficient. Multivariable analysis was carried out by the Cox proportional hazard model. Relation between biomarker levels (hPG_80_ and AFP) and other clinical variables were investigated using Logistic regression. Prism software (GraphPad Prism version 9.3.1 for Mac, GraphPad Software, La Jolla, CA, USA, www.graphpad.com) was used to perform all the statistical analyses and create figures. The level of significance was set at *p* < 0.05.

## 3. Results

### 3.1. Characteristics of the Study Population

**Figure 1:**
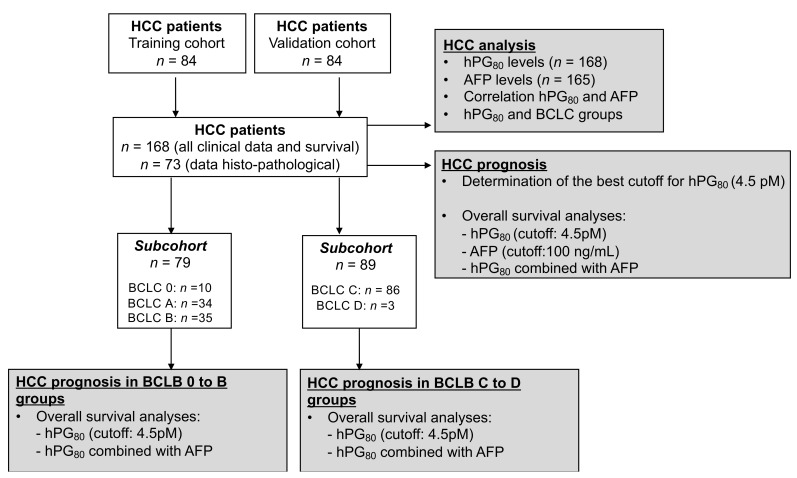
Study design.

A flowchart describing the main analyses performed in our study is shown on Figure 1. The demographic characteristics of the HCC and the various treatments are displayed in Table 1. The median patient age is 67 years and 88.7% were male. Cirrhosis was recorded in 138 patients (82.1%). Alcohol consumption was recorded in 107 patients (63.7%) and 31 patients (18.5%) were identified as having non-alcoholic steatohepatitis (NASH). Hepatitis B virus infections were found in 13 patients (7.7%) and hepatitis C virus infections were found in 45 patients (26.8%). According to the BCLC staging system, 6% had very early HCC (BCLC 0), 20.2% had early HCC (BCLC A), 20.8% had an intermediate HCC (BCLC B), 51.2% had an advanced HCC (BCLC C) and 1.8% had a terminal HCC (BCLC D) (Table 1). The median level in the HCC cohort of serum AFP is 40.2 ng/mL (Table 1). The median follow-up duration was 36 months, and the median overall survival after HCC diagnosis was 32 months. During the follow-up 96/168 (57%) patients died.

There was no correlation between hPG_80_ levels and age in this cohort (Spearman *r* = 0.0811, *p* = 0.296). Evaluation of hPG_80_ and AFP levels in all HCC patients revealed that there was no correlation between the two biomarkers (Spearman *r* = −0.0031, *p* = 0.968, Supplementary Figure S1A). There was a correlation between hPG_80_ and MELD and no correlation with creatinine levels in all HCC patients (Spearman *r* = 0.1704, *p* = 0.030 and r = 0.1271, *p* = 0.103, respectively, Supplementary Figure S1B,C).

hPG_80_ was detected in 141 of 168 (84%) HCC patients (threshold = 1 pM, corresponding to the limit of detection of the DxPG_80_ kit) with a median hPG_80_ concentration of 7.64 pM (IQR 2.53–28.28 pM) (Table 1). The optimal calculated hPG_80_ cutoff value across all 168 HCC patients was 4.5 pM and was used for all clinicopathological and prognosis analyses.

### 3.2. hPG_80_ and AFP Levels in the Training and Validation Cohorts

**Figure 2:**
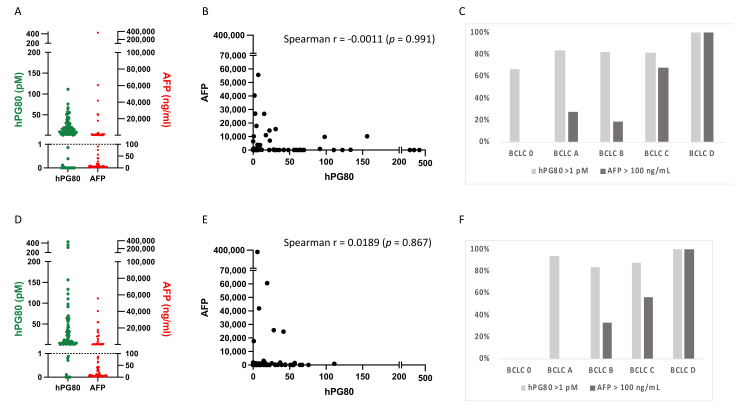
Characteristic of the training and validation cohorts. A to C, the training cohort. (**A**) Plasma hPG_80_ and AFP levels in HCC patients (*n* = 84). A cutoff value of 1 pM for hPG_80_ was set based on the limit of detection of hPG_80_. The cutoff value for AFP was set at 100 ng/mL. (**B**) Correlation between hPG_80_ and AFP levels in HCC patients was evaluated by the Spearman correlation coefficient. (**C**) hPG_80_ levels according to the BCLC stratification. D to F, the validation cohort. (**D**) Plasma hPG_80_ and AFP levels in HCC patients (*n* = 84). A cutoff value of 1 pM for hPG_80_ was set based on the limit of detection of hPG_80_. The cutoff value for AFP was set at 100 ng/mL. (**E**) Correlation between hPG_80_ and AFP levels in HCC patients was evaluated by the Spearman correlation coefficient. (**F**) hPG_80_ levels according to the BCLC stratification.

The demographic characteristics of the training and validation cohorts of HCC are displayed in the Supplementary Table S1. In the training cohort (84 patients), hPG_80_ was detected in 68 of 84 (81%) HCC patients (threshold = 1 pM, corresponding to the limit of detection of the DxPG_80_ kit). In the same cohort, AFP was detected in 38 of 84 (45.2%) HCC patients (threshold = 100 ng/mL) (Figure 2A). There was no correlation between hPG_80_ and AFP levels in the training cohort (Spearman *r* = −0.0011, *p* = 0.991, Figure 2B). hPG_80_ was detected in 21 of 27 (77.8%) HCC patients at early stages BCLC 0-A but only 6 (22.2%) were positive for AFP (Figure 2C).

These results were confirmed using a validation cohort (84 patients). hPG_80_ was detected in 73 of 84 (86.9%) HCC patients whereas AFP was detected in 34 of 84 (40.5%) HCC patients (Figure 2D). There was no correlation between hPG_80_ and AFP levels in the validation cohort (Spearman *r* = 0.0189, *p* = 0.867, Figure 2E). hPG_80_ was detected in 15 of 16 (93.8%) HCC patients at early stages BCLC 0-A whereas none were positive for AFP (Figure 2F).

Both cohorts behaved similarly and showed higher sensitivity of hPG_80_ compared to AFP, especially at early stages. Therefore, to increase statistical power, we pooled the training and validation cohorts to perform clinicopathological and prognosis analysis.

### 3.3. Correlation between hPG_80_ and AFP Levels and Various Clinicopathologic Features of HCC Patients According to Their Cutoff Values

We checked the association between plasma hPG_80_ levels and various clinicopathologic features of HCC patients according to the 4.5 pM cutoff value. As shown in Table 2, high hPG_80_ levels was correlated with vascular invasion (*p* = 0.0182), glutamine synthetase score (*p* = 0.0068), and inversely correlated with EpCAM expression (*p* = 0.0492) and with p53 and/or cytokeratine-19 (CK19) and/or EpCAM expression (*p* = 0.0088). These results suggest that hPG_80_ is strongly associated with prognosis factors of HCC. As a comparison, high AFP (>100 ng/mL) levels were associated with tumor size, vascular invasion, BCLC stages (*p* < 0.0001), and CRP (*p* = 0.0138) (Table 2). Thus, except for vascular invasion, these two biomarkers associate with distinct clinicopathologic features. We confirmed these results using regression analysis and calculation of the odds ratio (ORs) with the corresponding 95% CI (Table 3).

### 3.4. Prognosis Performance of hPG_80_ and AFP in HCC Patients

**Figure 3:**
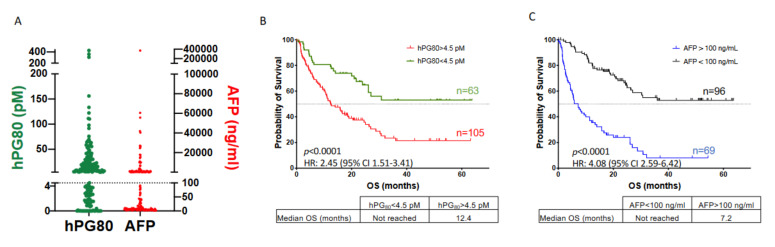
Levels of plasma hPG_80_ and overall survival of HCC patients according to hPG_80_ or AFP levels. (**A**) Plasma hPG_80_ and AFP levels in HCC patients (*n* = 168). A cutoff value of 4.5 pM for hPG_80_ was set based on the optimal calculated cutoff value. The cutoff value for AFP was set at 100 ng/mL. (**B**) Kaplan-Meier analysis of overall survival (OS) for HCC patients according to hPG_80_ levels (cutoff: 4.5 pM; hPG_80_ < 4.5 pM: *n* = 63; hPG_80_ > 4.5 pM: *n* = 105). (**C**) Kaplan-Meier analysis of OS for HCC patients according to AFP levels (cutoff: 100 ng/mL; AFP < 100 ng/mL: *n* = 96; AFP > 100 ng/mL: *n* = 69). The *p* values, hazard ratios (HR) and 95% confidence intervals are indicated.

We first investigated the prognostic value of hPG_80_ level by stratifying patients into high and low hPG_80_ levels based on the optimal calculated cutoff value of 4.5 pM. The 168 HCC patients were divided into two groups above and below this value: 63 patients displayed low hPG_80_ levels (hPG_80_−: <4.5 pM) and 105 patients displayed high hPG_80_ levels (hPG_80_+: >4.5 pM) (Figure 3A). Kaplan-Meier survival analysis showed that hPG_80_+ patients had a significantly shorter OS than hPG_80_− patients (12.4 months versus not reached; HR: 2.45, 95% CI: 1.51–3.41; *p* < 0.0001) (Figure 3B). As AFP has been demonstrated to be an independent predictor of OS in HCC patients, we also investigated the prognostic value of AFP in our cohort. AFP cutoff was set at 100 ng/mL based on the value used for liver transplantation [23]. HCC patients were categorized into two groups according to AFP concentration: 96 patients displayed low AFP levels (AFP−: <100 ng/mL) and 69 patients displayed high AFP levels (AFP+: >100 ng/mL) (Figure 3A). As shown in Figure 3C, the OS for AFP+ patients were as expected significantly shorter compared to AFP− patients (7.2 months versus not reached, HR: 4.08, 95% CI: 2.59–6.42; *p* < 0.0001). We performed the same analysis with an AFP cutoff value set at 20 ng/mL, which is a frequently used cutoff value for diagnosis or screening [24]. Patients with high AFP levels (>20 ng/mL, *n* = 89) had a significantly worse OS compared to patients with low AFP levels (<20 ng/mL *n* = 76) (10 months versus not reached; HR: 3.76, 95% CI: 2.49–5.7; *p* < 0.0001) (Supplementary Figure S2).

We then performed univariate and multivariable Cox regression analysis. Univariate analysis revealed that hPG_80_ levels were predictors for OS (hPG_80_ < 4.5 pM, HR = 0.38, 95% CI: 0.24–0.61; *p* < 0.0001). By multivariable analysis, hPG_80_ levels were found as an independent predictor for OS (hPG_80_ < 4.5 pM, HR = 0.48, 95% CI: 0.30–0.78; *p* = 0.003) (Table 4).

### 3.5. Prognosis Performance of hPG_80_ in Combination with AFP in HCC Patients

**Figure 4:**
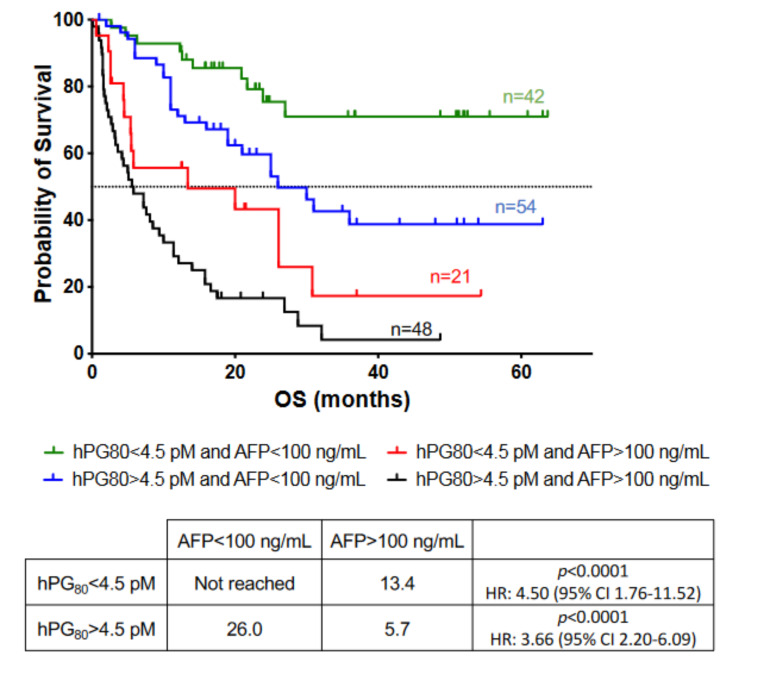
Overall survival of HCC patients based on combined hPG_80_ and AFP levels. Kaplan-Meier analysis of OS for HCC patients according to hPG_80_ and then to AFP levels (hPG_80_ < 4.5 pM and AFP < 100 ng/mL: *n* = 42; hPG_80_ < 4.5 pM and AFP > 100 ng/mL: *n* = 21; hPG_80_ > 4.5 pM and AFP < 100 ng/mL: *n* = 54, hPG_80_ > 4.5 pM and AFP > 100 ng/mL: *n* = 48).

Based on the results showing that plasma hPG_80_ is an independent prognostic biomarker for survival, we tested whether the combination of hPG_80_ and AFP could further improve prognostic stratification. We used hPG_80_ as primary criteria for our combined stratification analysis. HCC patients were categorized into four groups: hPG_80_−/AFP− (*n* = 42), hPG_80_−/AFP+ (*n* = 21), hPG_80_+/AFP− (*n* = 54) and hPG_80_+/AFP+ (*n* = 48) (Table 1). In the hPG_80_− group, AFP+ patients had a significantly worse prognosis than AFP- patients (13.4 months versus not reached, HR: 4.50, 95% CI: 1.76–11.52; *p* < 0.0001) (Figure 4). Correspondingly, in the hPG_80_+ group, AFP+ patients had a significantly worse prognosis than AFP− patients (26.0 months versus 5.7 months, HR: 3.66, 95% CI: 2.20–6.09; *p* < 0.0001) (Figure 4). Similar results were found when using 20 ng/mL as a cutoff for AFP (Supplementary Figure S3). Taken together, these results suggest an improved prognostic value when using combined hPG_80_ and AFP levels in HCC patients.

### 3.6. hPG_80_ and AFP Levels According to the BCLC Score in HCC Patients

The BCLC staging system is one of the most commonly used algorithms for prognosis of HCC patients [25]. The BCLC staging system classifies HCC patients into five categories (0, A, B, C, and D) based on four variables: tumor status, liver function, physical status, and cancer-related symptoms. We assessed the distribution of hPG_80_ levels according to the BCLC score.

**Figure 5:**
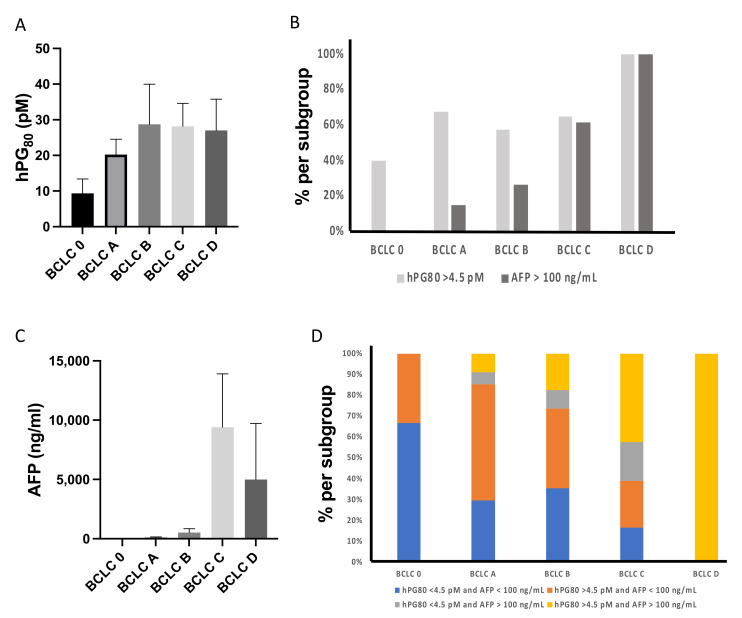
Detection rates for hPG_80_ and AFP in HCC patients with different BCLC stages. (**A**) hPG_80_ levels according to the BCLC stratification. (**B**) Percentage of HCC patients with high hPG_80_ levels (hPG_80_ > 4.5 pM) or high AFP levels (AFP > 100 ng/mL) according to BCLC stages. (**C**) AFP levels according to the BCLC stratification. (**D**) Percentage of HCC patients based on combined hPG_80_ and AFP levels and according to BCLC stages.

Our results showed that the levels of hPG_80_ and the distribution of hPG_80_+ samples are not statistically different among the different BCLC stages (*p* = 0.318, Figure 5A,B). On the other hand, and in accordance with previous studies [26], the percentage of AFP+ patients and the levels of AFP are significantly increased with higher BCLC stages (*p* < 0.0001, Figure 5B,C). It is worthy of note that, in BCLC 0 and A stages, 40% and 67.6% of patients have high hPG_80_ levels (>4.5 pM), whereas none and 14.7% have high AFP levels (>100 ng/mL), respectively (Figure 5B). These data highlight the potential value of combining hPG_80_ and AFP levels to increase detection and prognosis of HCC at early stages (for which the AFP rate is not increase in most of cases). Therefore, we further analyzed patient’s distribution among the different BCLC stages using combined hPG_80_ and AFP levels (Figure 5D). In the BCLC stage 0 and A group, 33% and 56% of patients were hPG_80_+/AFP−, whereas 0% and 6% of patients were hPG_80_−/AFP+, respectively. In the BCLC B and C groups, 38% and 22% of patients were hPG_80_+/AFP− whereas 9% and 19% of patients were hPG_80_−/AFP+, respectively. Finally, 100% of the patients in the BCLC stage D were hPG_80_+/AFP+.

### 3.7. Prognosis Performance of hPG_80_ and AFP According to the BCLC Score in HCC Patients

**Figure 6:**
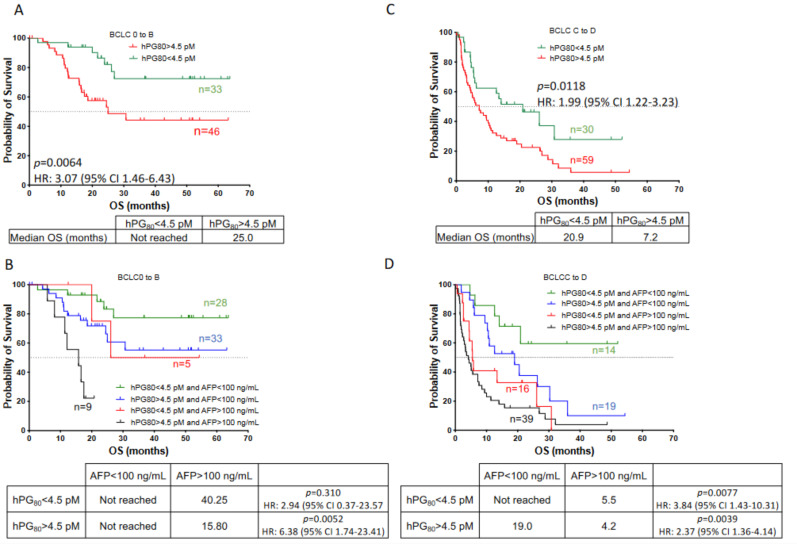
Overall survival of HCC patients with early to intermediate BCLC stages (BCLC 0 to B) and with advanced to terminal BCLC stages (BCLC C to D) according to hPG_80_ alone or in combination with AFP levels. (**A**) Kaplan-Meier analysis of OS for HCC patients with very early to intermediate BCLC stages (BCLC 0 to B, *n* = 79) according to hPG_80_ levels. Patients were divided into two groups based on hPG_80_ levels (cutoff: 4.5 pM; hPG_80_ < 4.5 pM: *n* = 33; hPG_80_ > 4.5 pM: *n* = 46). (**B**) Kaplan-Meier analysis of OS for HCC patients with BCLC 0 to B according to hPG_80_ and then to AFP levels (hPG_80_ < 4.5 pM and AFP < 100 ng/mL: *n* = 28; hPG_80_ < 4.5 pM and AFP > 100 ng/mL: *n* = 5; hPG_80_ > 4.5 pM and AFP < 100 ng/mL: *n* = 35, hPG_80_ > 4.5 pM and AFP > 100 ng/mL: *n* = 9). (**C**) Kaplan-Meier analysis of OS for HCC patients with advanced or terminal BCLC stages (BCLC C to D, *n* = 89) according to hPG_80_ levels. Patients were divided into two groups based on hPG_80_ levels (cutoff: 4.5 pM; hPG_80_ < 4.5 pM: *n* = 30; hPG_80_ > 4.5 pM: *n* = 59). (**D**) Kaplan-Meier analysis of OS for HCC patients with BCLC C to D according to hPG_80_ and then to AFP levels (hPG_80_ < 4.5 pM and AFP < 100 ng/mL: *n* = 14; hPG_80_ < 4.5 pM and AFP > 100 ng/mL: *n* = 16; hPG_80_ > 4.5 pM and AFP < 100 ng/mL: *n* = 19, hPG_80_ > 4.5 pM and AFP > 100 ng/mL: *n* = 39).

Next, we investigated the prognosis value of hPG_80_ alone or in combination with AFP in patients stratified by the BCLC classification. BCLC stratified patients were categorized into two groups: patients with very early to intermediate BCLC stages (BCLC 0 to B) (*n* = 79) and patients with advanced and terminal BCLC stages (BCLC C to D) (*n* = 89). The median OS was not reached in patients with BCLC stages 0 to B and 9.4 months in patients with BCLC stages C to D. As shown on Figure 6A, when BCLC stages 0 to B patients were divided into two subgroups based on hPG_80_ levels, the median OS for hPG_80_+ patients (*n* = 46) was significantly shorter than that of hPG_80_− patients (*n* = 33) (25 months versus not reached, HR: 3.07, 95% CI: 1.46–6.43; *p* = 0.0064). Likewise, AFP+ patients were significantly correlated with poorer OS (17.5 months versus not reached, HR: 3.16, 95% CI: 1.06–9.47; *p* = 0.0030) (Supplementary Figure S4A). Furthermore, we evaluated the prognostic significance of hPG_80_ in combination with AFP levels. Our data revealed that hPG_80_− patients with AFP > 100 ng/mL (*n* = 5) had a tendency of worse OS than those with AFP < 100 ng/mL patients (*n* = 28) (40.3 months versus not reached, HR: 2.94, 95% CI: 0.37–23.57; *p* = 0.310) (Figure 6B). Our data revealed also that hPG_80_+ patients with AFP > 100 ng/mL (*n* = 46) had a worse OS than that of patients with AFP < 100 ng/mL patients (*n* = 33) (15.8 months versus not reached, HR: 6.38, 95% CI: 1.74–23.41; *p* = 0.0052) (Figure 6B). Similar results were observed with an AFP cutoff value of 20 ng/mL (Supplementary Figure S5A,B).

Subsequently, we analyzed the prognosis value of hPG_80_ alone or in combination with AFP in patients with BCLC stages C to D. Again, we observed that the median OS for hPG_80_+ patients (*n* = 59) was significantly shorter than that of hPG_80_− patients (*n* = 30) (7.2 versus 20.9 months, HR: 1.99, 95% CI: 1.22–3.23; *p* = 0.0118) (Figure 6C). Likewise, AFP+ patients were significantly correlated with poorer OS (5.0 months versus 20.9 months, HR: 2.89, 95% CI: 1.79–4.67; *p* <0.0001) (Supplementary Figure S4B). Furthermore, we evaluated the prognostic significance of hPG_80_ in combination with AFP levels. hPG_80_− patients with AFP > 100 ng/mL (*n* = 16) had a worse OS than those with AFP < 100 ng/mL patients (*n* = 14) (5.5 months versus not reached, HR: 3.84, 95% CI: 1.43–10.31; *p* = 0.0077) (Figure 6D). Our data revealed also that hPG_80_+ patients with AFP > 100 ng/mL (*n* = 39) had a worse OS than AFP < 100 ng/mL patients (*n* = 19) (4.2 months versus 19.0 months, HR: 2.37, 95% CI: 1.36–4.14; *p* = 0.0039) (Figure 6D). Similar results, although not significant, were observed with an AFP cutoff value of 20 ng/mL (Supplementary Figure S5C,D). Taken together, our data suggest a promising prognostic value for hPG_80_ in HCC patients at all stages (also in early stages), and it improved stratification in combination with AFP.

## 4. Discussion

There is a clear need for biomarkers in HCC risk stratification, early detection, diagnosis, prognosis, and treatment response. In the present paper, we addressed the potential of a new blood biomarker, hPG_80_, as a prognostic factor in HCC patients. hPG_80_ has a tight relationship with cancer, with an already demonstrated example of its capacity to assess OS probability and be helpful for patients bearing metastatic renal cell carcinoma [13]. We now show that for HCC patients, hPG_80_ is also a potent prognostic factor to improve patient’s stratification either alone or in combination with AFP. hPG_80_ will be especially useful for AFP negative patients that are usually in the early stages and therefore curable.

Since 84% of the patients have detectable levels of hPG_80_ (>1 pM), and only 41% for AFP (above 100 ng/mL), we decided to use hPG_80_ as primary criteria for our combined stratification analysis. Interestingly, the percentage of non-detected HCC patients (i.e., 16%) is consistent to what we observed in other types of cancers suggesting the possibility of a small fraction of low hPG_80_ expressing tumors [9,13].

In patients with low hPG_80_ level, AFP further stratifies patient population with much shorter OS (13.4 months) when AFP level is high that those patients with low AFP level (not reached). The same also applies for patients with high hPG_80_ at diagnostic. AFP further stratifies patients with a better OS if AFP level is low (26 months versus 5.7 months respectively).

In line with the above analysis, hPG_80_ can stratify HCC patients even at early stage. For instance, in BCLC 0-A patients, AFP is only high in 11.4% of those patients in comparison with hPG_80_, which is high in 59.1% of them. This allows a better stratification of the patients with an OS probability switching from not reached to 25 months when hPG_80_ levels are high. Thus, what was already relevant for the entire patient population is even more relevant when patients are classified according to BCLC scores. Therefore, in the case of liver transplantation, we could evaluate if hPG_80_ levels could improve the AFP-score built by the Liver Transplant French Study Group based on AFP level, number of nodules and the size of the largest nodule to identify candidates with low risk of HCC recurrence or who will survive for 5 years after liver transplantation [27]. We are currently setting up a clinical study to evaluate this hypothesis.

Early stages of HCC are characterized by dysplastic lesions, frequently arising in chronic inflammatory liver disease or hepatitis that contributes to fibrosis and, subsequently, cirrhosis affecting the liver function and often leading to patient death [4]. Liver fibrosis remains therefore a major health problem with a high mortality rate predisposing to HCC. Around 90% of HCC cases develop in a background of cirrhosis, but less than 5% of patients with cirrhosis progress to HCC annually [28]. We are currently setting up a clinical study to evaluate if an increase of hPG_80_ in cirrhotic patients could identify the ones who will develop an HCC.

There are several other biomarkers under investigation that also showed a potential ability to establish a prognosis for HCC patients. Laminin-γ2 has been recently demonstrated the ability to identify those patients that will develop metastasis, thus having poor outcomes [29]. The results concerning Laminin-γ2 expression are interesting and seem to be associated to S1 [30] or G3 [31] of the HCC biological classification. In our study, there was a positive correlation between hPG_80_ levels and histologic features (GS staining, pseudoglandular score and the steatohepatitis score) classically associated with S3 or G4–6 subgroups of these classifications underlying the potential benefit to build a multi biomarker tool testing AFP, Laminin-γ2 and hPG_80_ levels. Currently, only serum AFP levels have enough high-level supporting evidence to be used as a biomarker in clinical practice to predict prognosis of HCC patients. But the disagreement between different international guidelines in terms of the AFP threshold for HCC diagnosis and prognosis has been continued for several decades, and it has not yet been resolved so far. In our trial we decide to retain two AFP cutoff values: 20 ng/mL, which is a frequently used cutoff value for diagnosis and screening, and 100 ng/mL used in France to identify candidates with low risk of HCC recurrence or death in national liver transplantation scores [27]. Considering circulating tumor DNA (ctDNA) and cell-free DNA (cfDNA), there are numerous studies showing that they can also serve as prognostic factors, in particular in terms of recurrence occurrence when still detectable after treatment [32].

Although the present study provides an important potential clinical utility for hPG_80_, it has some limitations. It is a prospectively enrolled HCC cohort which was retrospectively analysed. In the cohort, alcohol consumption was recorded in a majority of patients as it was already reported in a national French cohort [33]. However, among these 107 patients, 48 also had other potential factors of hepatopathy (diabetes, overweight, dyslipidemia…). Patients received various treatments: chemoembolization, radiofrequency, targeted therapies or immunotherapies. Although hPG_80_ showed OS prognostic value whatever the treatment, it might be important to perform the analysis treatment by treatment in order to gain insight into the management of HCC patients.

## 5. Conclusions

In conclusion, plasma hPG_80_ alone or in combination with AFP improves prognosis evaluation of OS in HCC patients. hPG_80_ is easily detectable in the plasma and could be tested throughout the patient’s journey to potentially identify patients who may need a deeper biological assessment at an acceptable economic cost. Further ongoing studies will warrant that the evaluation of hPG_80_ might be proposed as an additional tool for the benefit of cancer patients.

## Figures and Tables

**Table 1:** Clinical and pathological characteristics for HCC patients and control cohorts.

Cohort		HCC N (%) *n* = 168
**Age, years**	Median (range)	67 (27–85)
**Gender**	Male	149 (88.7%)
	Female	19 (11.3%)
**Laboratory values**		
**hPG_80_**	Median (IQR), pM	7.64 (2.53–28.28)
	Mean (SE), pM	25.52 (4.16)
**AFP**	Median (IQR), ng/mL	40.20 (5.55–712.50)
	Mean (SE), ng/mL	5066 (2340)
**Etiology**		
**NASH**	Y	31 (18.5%)
	N	137 (81.5%)
**Alcohol consumption**	Y	107 (63.7%)
	N	61 (36.3%)
**Hepatitis B virus**	Y	13 (7.7%)
	N	155 (92.3%)
**Hepatitis C virus**	Y	45 (26.8%)
	N	123 (73.2%)
**Tumor size**	<3 cm	54 (49.1%)
	3 to 5 cm	23 (20.9%)
	>5 cm	33 (30.0%)
**Vascular invasion**	Y	57 (33.9%)
	N	108 (64.3%)
	Unspecified	3 (1.8%)
**Cirrhosis**	Y	138 (82.1%)
	N	30 (17.9%)
**BCLC**	0	10 (6.0%)
	A	34 (20.2%)
	B	35 (20.8%)
	C	86 (51.2%)
	D	3 (1.8%)
**hPG_80_ (cutoff: 4.5 pM) and AFP (cutoff: 100 ng/mL) levels**	neg/neg	42 (25.5%)
	neg/pos	21 (12.7%)
	pos/neg	54 (32.7%)
	pos/pos	48 (29.1%)
**Treatments**	Radiofrequency	20 (11.9%)
	Surgery	5 (3.0%)
	Transplantation	8 (4.7%)
	TACE	28 (16.7%)
	SIRT	6 (3.6%)
	Sorafenib	50 (29.7%)
	Others TKI	10 (6.0%)
	Gemox	2 (1.2%)
	Immunotherapy	20 (11.9%)
	Supportive care	19 (11.3%)

Liver Center, NASH: nonalcoholic steatohepatitis, GEMOX: Gemcitabine + Oxaliplatine, TACE: Transarterial chemoembolization, SIRT: selective internal radiation therapy TKI: tyrosine kinase inhibitors.

**Table 2:** hPG_80_ and AFP levels co-variables analysis.

hPG_80_ < 4.5 pM			hPG_80_ > 4.5 pM	*p*	AFP < 100 ng/mL	AFP > 100 ng/mL	*p*
Alcohol	Y	35	72	0.0996	66	40	0.1882
	N	28	33		30	29	
HCV	Y	15	30	0.5904	28	16	0.4761
	N	48	75		68	53	
NASH	Y	11	20	0.2568	14	17	0.1029
	N	52	85		82	52	
Tumor size	<3 cm	22	29	0.1193	44	7	<0.0001
	3 to 5 cm	10	13		19	4	
	>5 cm	8	27		15	19	
Vascular invasion	Y	14	42	0.0182	17	39	<0.0001
	N	48	61		78	28	
AFP score	0	13	14	0.6905	37	0	<0.0001
	1	6	8		14	0	
	2	4	10		10	4	
	>3	7	10		8	9	
CRP	mean (sd)	15.7 (34.8)	26.2 (38.5)	0.1359	15.6 (31.4)	32.6 (43)	0.0138
	Median (range)	4 (0.3–212)	9.7 (0.5–210.0)		5.2 (0.3–212)	14.7 (0.5–210)	
BCLC	0	6	4	0.3183	9	0	<0.0001
	A	12	22		29	5	
	B	15	20		25	9	
	C	30	56		35	52	
	D	0	3		0	3	
Tumor differentiation	Poor	6	10	0.0529	11	5	0.6533
	intermediate	32	30		39	22	
	good	3	13		8	7	
GS Score	0–3	15	6	0.0068	4	6	0.3015
	4–6	19	33		38	24	
CK19	pos	2	4	0.6787	4	2	0.6654
	neg	32	35		38	28	
p53	pos	13	9	0.1591	11	11	0.3414
	neg	21	30		31	19	
EPCAM	pos	5	1	0.0492	3	3	0.7895
	neg	14	21		19	15	
p53 or CK19 or EPCAM	pos	12	5	0.0088	9	10	0.3561
	neg	7	17		13	8	

**Table 3:** hPG_80_ and AFP levels odds ratio analysis.

Characteristics	hPG_80_ > 4.5 pM		AFP > 100 ng/mL	
	Odds Ratio	95% CI	Odds Ratio	95% CI
Alcohol	1.745	0.915–3.340	0.627	0.328–1.194
HCV	1.280	0.631–2.674	0.733	0.354–1.480
NASH	1.112	0.501–2.578	1.915	0.872–4.266
Tumor size				
<3 cm	0.986	0.365–2.706	1.105	0.268–3.979
3 to 5 cm	2.655	1.044–7.268	5.559	2.096–15.915
Vascular invasion	2.361	1.177–4.941	6.391	3.179–13.344
AFP score				
1	1.238	0.338–4.697		
2	2.321	0.608–10.189		
>3	1.327	0.391–4.649		
BCLC				
B	0.923	0.374–2.282	2.736	0.844–9.808
C-D	1.362	0.642–2.865	12.667	4.898–39.672
Tumor differentiation				
intermediate	0.563	0.173–1.705	1.241	0.395–4.360
good	2.600	0.543–14.854	1.925	0.450–8.768
GS Score 4–6	4.342	1.499–13.966	0.933	0.334–2.662
p53	0.485	0.171–1.326	1.632	0.591–4.543

**Table 4:** hPG_80_ multivariate Cox analysis.

OS			
	Hazard Ratio	95% CI	*p*
**hPG_80_**	0.48	0.30–0.78	0.003
**AFP**	1.40	0.91–2.15	0.121
**BCLC**			
A	0.71	0.18–2.78	0.623
B	1.73	0.50–5.94	0.383
C	4.72	1.46–15.25	0.009
D	65.42	10.81–396.11	<0.001
**Age**	1.03	1.01–1.05	0.013
**Gender**	4.54	1.71–12.03	0.002

## Data Availability

The data presented in this study are available on request from the corresponding author.

## Supplementary Materials

The following supporting information can be downloaded at: https://www.mdpi.com/article/10.3390/cancers14020402/s1. Figure S1. Scatter plots of hPG_80_ and AFP, MELD and creatinine in HCC patients. Figure S2. Overall survival of HCC patients according to AFP levels. Kaplan-Meier analysis of OS for HCC patients according to AFP levels (cutoff: 20 ng/mL; AFP < 20 ng/mL: *n* = 76; AFP > 20 ng/mL: *n* = 89). Figure S3. Overall survival of HCC patients based on combined hPG_80_ and AFP levels. Figure S4. Overall survival of HCC patients depending on the BCLC stages according to AFP levels. Figure S5. Overall survival of HCC patients with early to intermediate BCLC stages (BCLC 0 to B) and with advanced to terminal BCLC stages (BCLC C to D) according to hPG_80_ alone or in combination with AFP levels at 20 ng/mL. Table S1. Clinical and pathological characteristics for the training and validation cohorts.
