# Supplementary material for: Plasma hPG80 (Circulating Progastrin) as a Novel Prognostic Biomarker for Hepatocellular Carcinoma
Source: Cancers (Basel). 2022 Jan 13;14(2):402. doi: 10.3390/cancers14020402 (PMC8774261; doi:10.3390/cancers14020402)
Supplement: Supplementary file 1 [file cancers-14-00402-s001.zip › cancers-1551292-supplementary.pdf]

cancers

MDPI

Supplementary Materials

Plasma hPG80 (Circulating Progastrin) as a Novel Prognostic Biomarker for Hepatocellular Carcinoma

Marie Dupuy [1], Sarah Iltache [1], Benjamin Rivière [1], Alexandre Prieur [2], George Philippe Pageaux [1], José Ursic Bedoya [1], Stéphanie Faure [1], Heloïse Guillaumée [1] and Eric Assenat [1,*]

[1] Department of Medical Oncology, CNRS UMR 5535 St-Eloi University Hospital Montpellier, School of Medicine, 34295 Montpellier, France; m-dupuy@chu-montpellier.fr (M.D.); sarah.iltache@gmail.com (S.I.); b-riviere@chu-montpellier.fr (B.R.); gp-pageaux@chu-montpellier.fr (G.P.P.); j-ursicbedoya@chu-montpellier.fr (J.U.B.); s-faure@chu-montpellier.fr (S.F.); h-guillaumee@chu-montpellier.fr (H.G.)

[2] ECS-Progastrin, 1004 Lausanne, Switzerland; a.prieur@ecs-progastrin.com

* Correspondence: e-assenat@chu-montpellier.fr

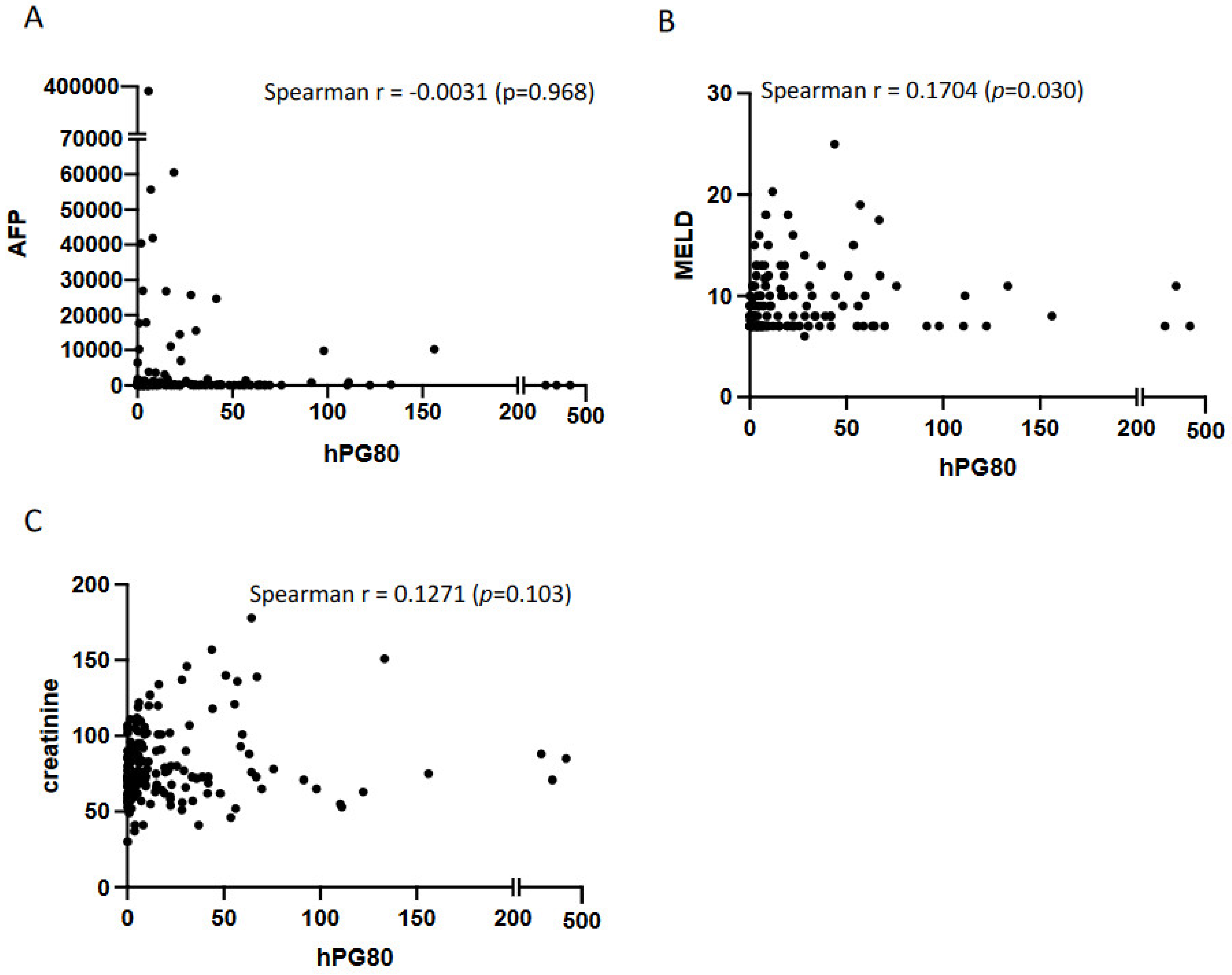

Figure S1. Scatter plots of hPG80 and AFP, MELD and creatinine in HCC patients. (**A**) Correlation between hPG80 and AFP levels in HCC patients was evaluated by the Spearman correlation coefficient. (**B**) Correlation between hPG80 and MELD levels in HCC patients was evaluated by the

Spearman correlation coefficient. (**C**) Correlation between hPG80 and creatinine levels in HCC patients was evaluated by the Spearman correlation coefficient.

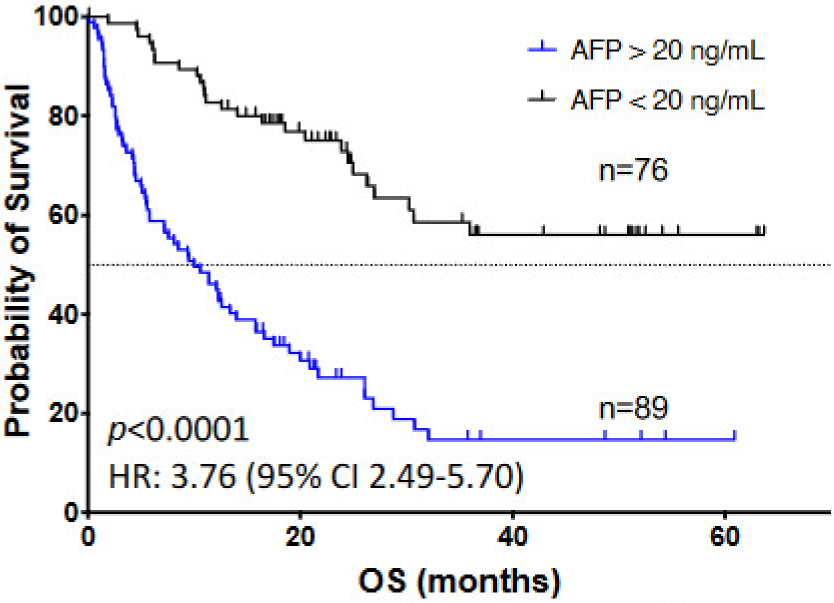

	AFP < 20 ng/ml	AFP > 20 ng/ml
Median OS (months)	Not reached	10.0

Figure S2. Overall survival of HCC patients according to AFP levels. Kaplan-Meier analysis of OS for HCC patients according to AFP levels (cutoff: 20 ng/mL; AFP < 20 ng/mL: n = 76; AFP > 20 ng/mL: n = 89). The p values, hazard ratios (HR) and 95% confidence intervals are indicated.

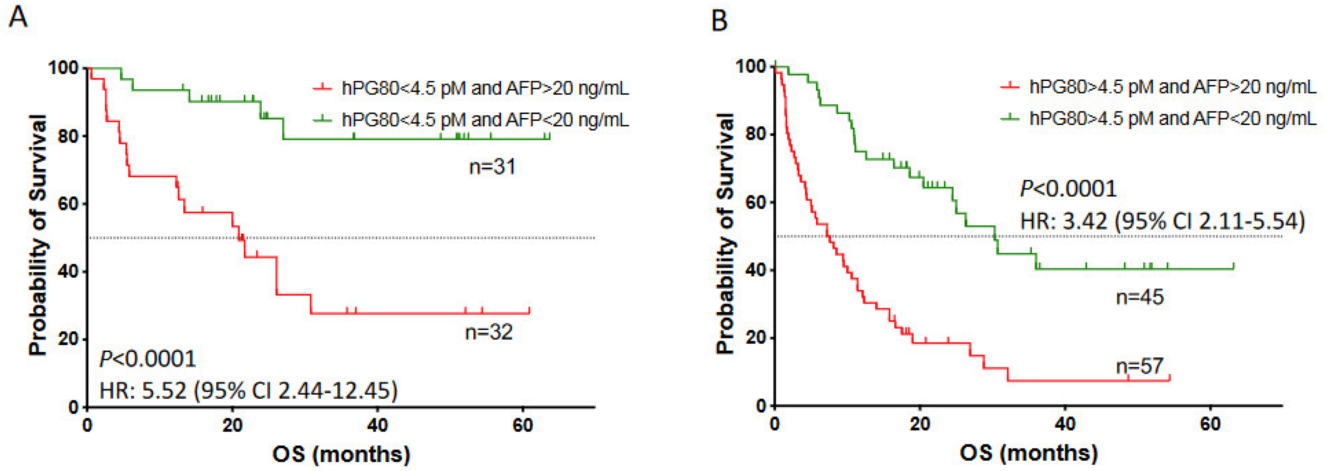

	AFP<20 ng/mL	AFP>20 ng/mL
Median OS (months)	Not reached	20.9

	AFP<20 ng/mL	AFP>20 ng/mL
Median OS (months)	30.30	7.60

Figure S3. Overall survival of HCC patients based on combined hPG80 and AFP levels. (**A**) Kaplan-Meier analysis of OS for HCC patients with low hPG80 levels (hPG80 < 4.5 pM; n = 63) according to AFP levels (cutoff: 20 ng/mL; AFP < 20 ng/mL: n = 31; AFP > 20 ng/mL: n = 32). (**B**) Kaplan-Meier

analysis of OS for HCC patients with high hPG80 levels (hPG80 > 4.5 pM; *n* = 102) according to AFP levels (cutoff: 20 ng/mL; AFP < 20 ng/mL: *n* = 45; AFP > 20 ng/mL: *n* = 57). The *p* values, hazard ratios (HR) and 95% confidence intervals are indicated.

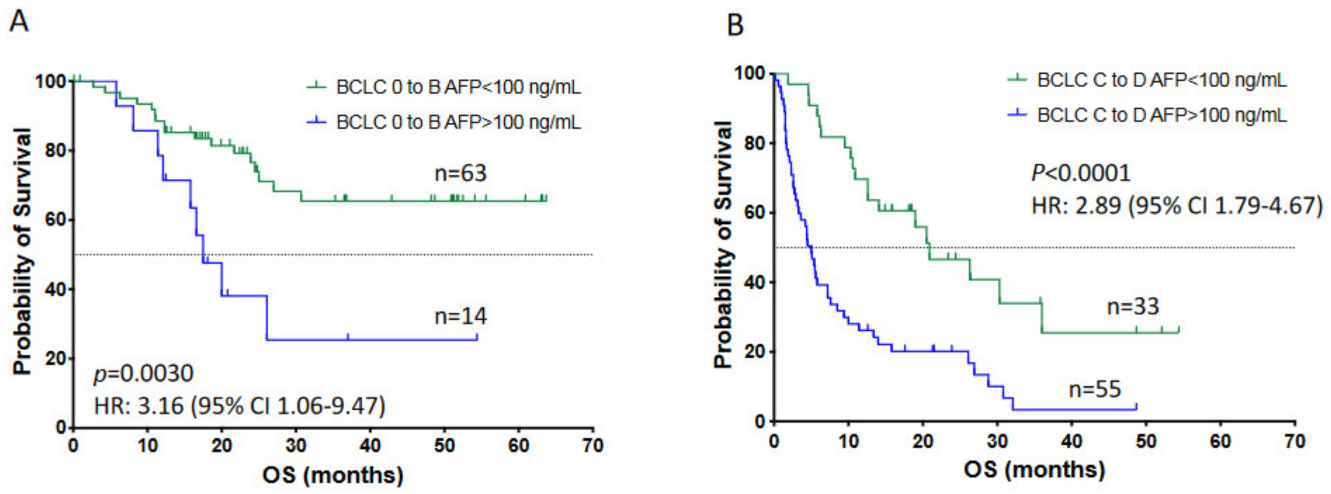

	AFP < 100 ng/ml	AFP > 100 ng/ml
Median OS (months)	Not reached	17.5

	AFP < 100 ng/ml	AFP > 100 ng/ml
Median OS (months)	20.9	5.0

Figure S4. Overall survival of HCC patients depending on the BCLC stages according to AFP levels. (**A**) Kaplan-Meier analysis of OS for HCC patients with very early to intermediate BCLC stages (BCLC 0 to B, *n* = 77) according to AFP levels. Patients were divided into two groups based on AFP levels (cutoff: 100 ng/mL; AFP < 100 ng/mL: *n* = 63; AFP > 100 ng/mL: *n* = 14). (**B**) Kaplan-Meier analysis of OS for HCC patients with advanced to terminal BCLC stages (BCLC C to D, *n* = 88) according to AFP levels. Patients were divided into two groups based on AFP levels (cutoff: 100 ng/mL; AFP < 100 ng/mL: *n* = 33; AFP > 100 ng/mL: *n* = 55).

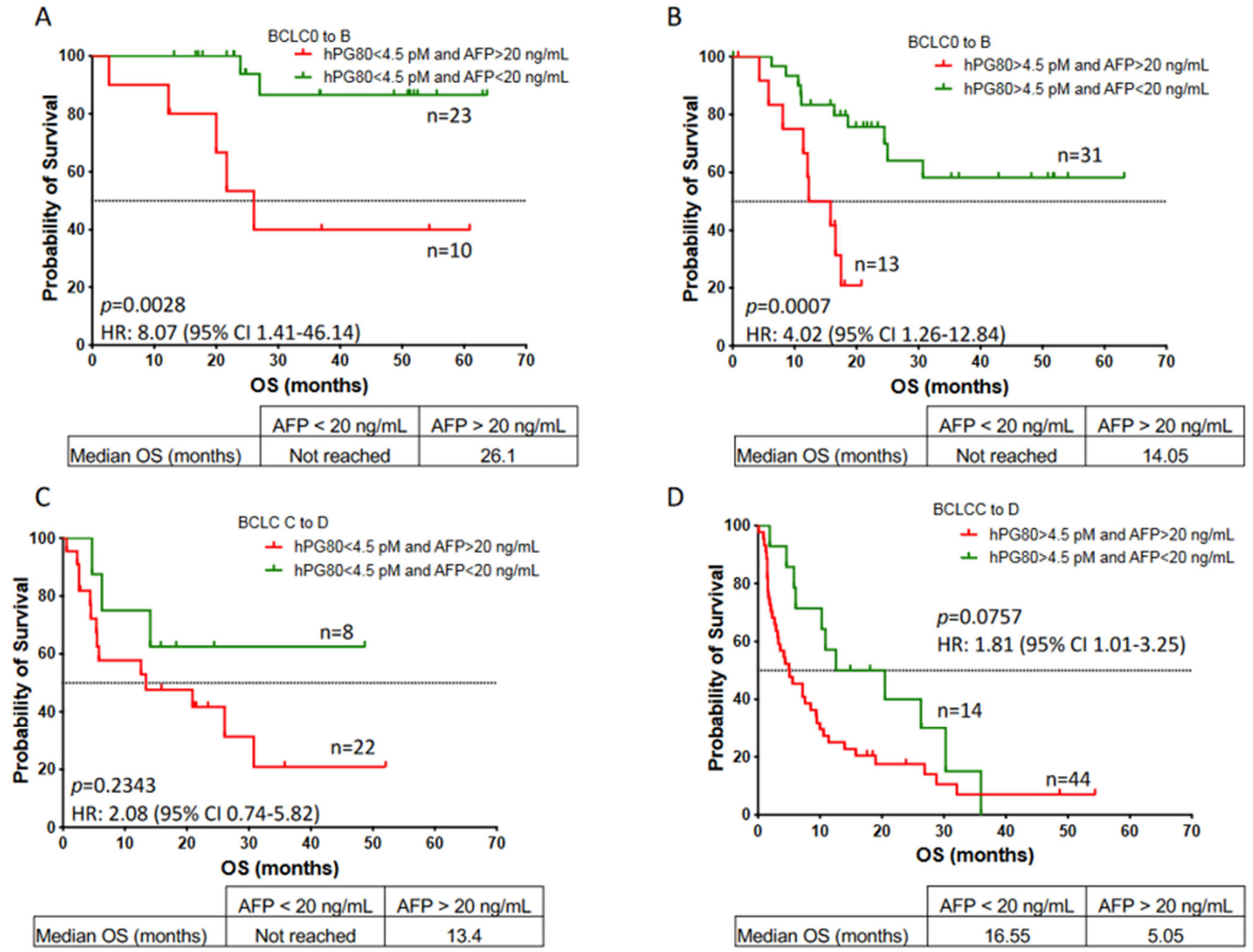

	AFP < 20 ng/mL	AFP > 20 ng/mL
Median OS (months)	Not reached	26.1

	AFP < 20 ng/mL	AFP > 20 ng/mL
Median OS (months)	Not reached	14.05

	AFP < 20 ng/mL	AFP > 20 ng/mL
Median OS (months)	Not reached	13.4

	AFP < 20 ng/mL	AFP > 20 ng/mL
Median OS (months)	16.55	5.05

Figure S5. Overall survival of HCC patients with early to intermediate BCLC stages (BCLC 0 to B) and with advanced to terminal BCLC stages (BCLC C to D) according to hPG_{80} alone or in combination with AFP levels at 20 ng/mL. (**A**) Kaplan-Meier analysis of OS for HCC patients with BCLC 0 to B and low hPG_{80} levels (hPG_{80} < 4.5 pM) (n = 33) according to AFP levels. Patients were divided into two groups based on AFP levels (cutoff: 20 ng/mL; AFP < 20 ng/mL: n = 23; AFP > 20 ng/mL: n = 10). (**B**) Kaplan-Meier analysis of OS for HCC patients with BCLC 0 to B and high hPG_{80} levels (hPG_{80} > 4.5 pM) (n = 44) according to AFP levels. Patients were divided into two groups based on AFP levels (cutoff: 20 ng/mL; AFP < 20 ng/mL: n = 31; AFP > 100 ng/mL: n = 13). (**C**) Kaplan-Meier analysis of OS for HCC patients with BCLC C to D and low hPG_{80} levels (hPG_{80} < 4.5 pM) (n = 30) according to AFP levels. Patients were divided into two groups based on AFP levels (cutoff: 20 ng/mL; AFP < 20 ng/mL: n = 8; AFP > 20 ng/mL: n = 22). (**D**) Kaplan-Meier analysis of OS for HCC patients with very BCLC C to D and high hPG_{80} levels (hPG_{80} > 4.5 pM) (n = 58) according to AFP levels. Patients were divided into two groups based on AFP levels (cutoff: 20 ng/mL; AFP < 20 ng/mL: n = 14; AFP > 20 ng/mL: n = 44). The p values, hazard ratios (HR) and 95% confidence intervals are indicated.

Table S1. Clinical and pathological characteristics for the training and validation cohorts.

		Training Cohort N (%) *n* = 84	Validation Cohort N(%) *n* = 84
**Age, years**	Median (range)	66 (27–85)	68 (45–84)
**Gender**	Male	74 (88.1%)	75 (89.3%)
	Female	10 (11.9%)	9 (10.7%)
**Laboratory values**			
**hPG_{80}**	Median (IQR), pM	8.79 (2.93–21.94)	6.31 (2.40–31.85)
	Mean (SE), pM	16.77 (2.26)	34.27 (7.92)
**AFP**	Median (IQR), ng/mL	27.80 (5.00–825.50)	41.05 (6.39–479.50)
	Mean (SE), ng/mL	7006 (4663)	3196 (975)
**Etiology**			
**NASH**	Y	9 (10.7%)	22 (26.2%)
	N	75 (89.3%)	62 (73.8%)
**Alcohol consumption**	Y	52(61.9%)	55 (65.5%)
	N	32 (38.1%)	29 (34.5%)
**Hepatitis B virus**	Y	8 (9.5%)	5 (6.0%)
	N	76 (90.5%)	79 (94.0%)
**Hepatitis C virus**	Y	28 (33.3%)	17 (20.2%)
	N	56 (66.7%)	67 (79.8%)
**Cirrhosis**	Y	73 (86.9%)	65 (77.4%)
	N	11 (13.1%)	19 (22.6%)
**BCLC**	0	9 (10.7%)	1 (1.25%)
	A	18 (21.4%)	16 (19.0%)
	B	17 (20.2%)	18 (21.4%)
	C	38 (45.2%)	48 (57.1%)
	D	2 (2.5%)	1 (1.25%)

Abbreviations, AFP: alpha-fetoprotein; IQR: interquartile range; SE: standard error; BCLC: Barcelona Clinic Liver Center; NASH: nonalcoholic steatohepatit.
